# Where and When Should Sensors Move? Sampling Using the Expected Value of Information

**DOI:** 10.3390/s121216274

**Published:** 2012-11-26

**Authors:** Sytze de Bruin, Daniela Ballari, Arnold K. Bregt

**Affiliations:** 1 Laboratory of Geo-Information Science and Remote Sensing, Wageningen University, P.O. Box 47, 6700 AA Wageningen, The Netherlands; E-Mail: arnold.bregt@wur.nl; 2 Instituto de Estudios de Régimen Seccional del Ecuador, Azuay University, 24 de Mayo 7-77 and Hernán Malo, Cuenca, EC010150, Ecuador; E-Mail: dballari@uazuay.edu.ec

**Keywords:** iterative sampling, adaptive sampling, infill sampling, decision analysis, environmental monitoring, geostatistics, mobile sensors

## Abstract

In case of an environmental accident, initially available data are often insufficient for properly managing the situation. In this paper, new sensor observations are iteratively added to an initial sample by maximising the global expected value of information of the points for decision making. This is equivalent to minimizing the aggregated expected misclassification costs over the study area. The method considers measurement error and different costs for class omissions and false class commissions. Constraints imposed by a mobile sensor web are accounted for using cost distances to decide which sensor should move to the next sample location. The method is demonstrated using synthetic examples of static and dynamic phenomena. This allowed computation of the true misclassification costs and comparison with other sampling approaches. The probability of local contamination levels being above a given critical threshold were computed by indicator kriging. In the case of multiple sensors being relocated simultaneously, a genetic algorithm was used to find sets of suitable new measurement locations. Otherwise, all grid nodes were searched exhaustively, which is computationally demanding. In terms of true misclassification costs, the method outperformed random sampling and sampling based on minimisation of the kriging variance.

## 1. Introduction

In case of calamities such as the major Fukushima Daiichi nuclear power plant accident in Japan [1] or a recent fire at a chemical plant in The Netherlands during which large quantities of polycyclic aromatic hydrocarbons were released, authorities have to quickly decide whether or not people living in the vicinity of the source of pollution have to be evacuated. After the incident, field crops grown in the area affected by deposited pollutants may have to be discarded because they are unsuitable for human consumption, while soil remediation may be needed to allow future cultivation [2]. Since accidents are rare, usually there is no dense monitoring network in place so that decision making has to rely on information initially obtained from a small sample. This situation may improve when non-covered regions are “filled in” by additional sampling [3,4] e.g., using mobile sensors.

The consequences of decisions made about local safety are costly. On the one hand, evacuating and cleaning sites are expensive tasks and these costs can be avoided whenever locations are safe. On the other hand, not cleaning or evacuating unsafe areas will put the population at risk, which typically involves even higher costs at later stages. Thus, the problem faced is twofold: (1) deciding between safe and unsafe areas and (2) deciding about when and where to sample so that that the obtained data optimally support decision making.

In contrast to [5,6], who aimed to find targets within a search area, our objective is to create a complete map of some environmental variable over a study area. Given this objective, our focus is on model-based rather than design-based approaches [7]. While there is a wealth of publications on (adaptive) spatial sampling for mapping, most evaluation criteria used so far did not consider the sample optimisation problem in direct connection to the decision problem (1) listed above. Typically, optimisation has focused on the quality of the estimated covariance function [8–10], minimisation of the variance of the prediction error [8,11,12] or it used some information entropy based criterion [13–15]. Note that the latter criteria depend on the distance between data points rather than the data values acquired, while criteria related to the covariance function only indirectly depend on the data values.

Recent exceptions are [16–18]. The criterion used by Peyrard *et al.*[18] implicitly assumes equal costs for class omissions (false negatives) and false class commissions (false positives). Heuvelink *et al.*[16] assigned unequal costs to the two types of errors but their method requires extensive Monte Carlo simulation for computing the expected costs associated with sampling designs within an iterative procedure for searching the optimal set of sample locations. This renders the method computationally very demanding. In contrast, Ballari *et al.*[17] employed indicator kriging for computing probability maps of presence (above critical threshold) and absence (below critical threshold) of some environmental variable and applied the concept of expected value of information (EVOI) [19–21] to decide upon the best sampling locations. EVOI expresses the benefit expected from data collection prior to actually doing the measurements [22–24]. Computational complexity in this case mainly involves the search for the best locations while the computation of the expected costs is relatively fast.

While the work of Ballari *et al.*[17] demonstrated the use of EVOI assessing a static phenomenon using measurements of a single sensor per time step, this paper aims to extend their methods by: (1) allowing for simultaneous measurement by multiple sensors and (2) supporting the mapping of spatio-dynamic fields. We first describe the EVOI method including its extensions and underlying geostatistical interpolation and next demonstrate and evaluate it using synthetic data sets.

## 2. Methodology

### 2.1. Expected Value of Information

**Figure 1.**
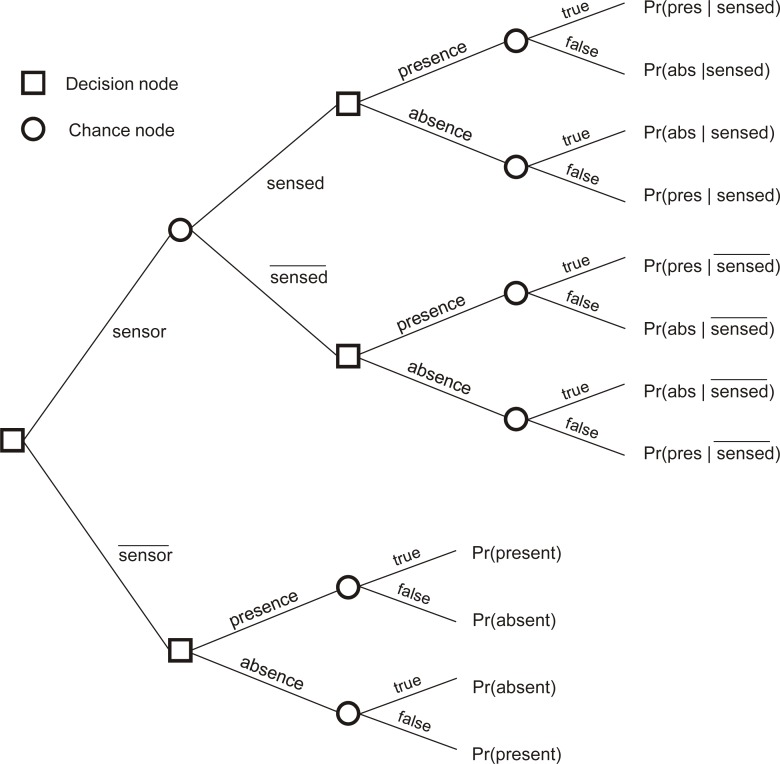
Decision tree showing decisions to place a sensor (sensor) or not ( $\bar{\text{sensor}}$) and to map presence or absence of a phenomenon (e.g., concentration pollutant exceeds a critical threshold).

EVOI is estimated as the difference between expected costs at the present stage of knowledge and expected costs when new information becomes available [19–21]. Figure 1 shows a decision tree with square nodes indicating decisions for measuring the phenomenon by putting a sensor at some location and decisions on whether to map presence or absence of the phenomenon using the information at hand. It concerns local EVOI, *i.e.*, it concerns a single location on the map. Chance nodes (circles) indicate the outcome of random events once a decision has been made. For example, if a measurement is made, the device may indicate presence (*sensed*) or absence $\left(\bar{\mathrm{sensed}}\right)$ of the phenomenon. The probability of obtaining a positive sensor signal at the location, Pr(*sensed*), is computed from sensor specifications and the prior probability of presence and absence using Equation (1):

(1) Pr(sensed)=Pr(sensed|present)×Pr(present)+Pr(sensed|absent)×Pr(absent)

where Pr(*sensed* | *present*) is the probability that a the sensor correctly gives a positive signal if the phenomenon is above the critical threshold and $\text{Pr}(\mathrm{sensed}|\mathrm{absent})=1-\text{Pr}(\bar{\mathrm{sensed}}|\mathrm{absent})$ is the probability that the sensor gives a false warning. The latter probabilities are assumed to be documented in the sensor specifications (*i.e.*, sensitivity and specificity of the sensor) and they are useful for representing measurement error. The prior probabilities Pr(*present*) and Pr(*absent*) are computed from previous data, starting from an initial sample and accounting for conditional dependence between data points, as explained in the next section. Formally we could thus also use the notation Pr(*present* | *previous data*). These probabilities are subjective in the sense that they depend on the acquired data as well as on the computational method and its parameters (see Section 4.1).

In this work, decision making was assumed to be based on Bayes actions, *i.e.*, minimisation of expected loss. In other words, we assume rational decision making. Measurement with a sensor thus only makes sense if the expected loss of the upper branch of Figure 1 is lower than the expected loss of the lower branch (for now we neglect the costs of measurements). If only misclassifications involve costs, the expected cost of the lower branch is calculated by Equation (2):

(2) $$E({\mathrm{cost}}_{\mathrm{lower}})=\text{min}({\mathrm{cost}}_{\mathrm{false positive}}\times \text{Pr}(\mathrm{absent}),{\mathrm{cost}}_{\mathrm{false negative}}\times \text{Pr}(\mathrm{present})$$

where min(.) is a function returning the minimum of its arguments and *cost_false negative_* and *cost_false positive_* are the costs of misclassification. The conditional probabilities shown in Figure 1 are calculated with Bayes’ rule, as in Equation (3) for example:

(3) $$\text{Pr}(\mathrm{absent}|\mathrm{sensed})=\frac{\text{Pr}(\mathrm{sensed}|\mathrm{absent})\times \text{Pr}(\mathrm{absent})}{\text{Pr}(\mathrm{sensed})}$$

Hence, the expected cost of the upper branch was calculated by Equation (4):

(4) $$\begin{array}{l} E({\mathrm{cost}}_{\mathrm{upper}})=\text{Pr}(\mathrm{sensed})\\ \;\;\;\;\;\;\;\;\;\;\;\;\;\;\;\;\;\;\;\;\;\;\;\;\;\;\;\;\;\;\;\;\;\;\;\;\;\;\;\;\;\;\;\;\;\;\;\;\;\;\;\times \text{min}({\mathrm{cost}}_{\mathrm{false positive}}\times \text{Pr}(\mathrm{absent}|\mathrm{sensed}),{\mathrm{cost}}_{\mathrm{false negative}}\\ \;\;\;\;\;\;\;\;\;\;\;\;\;\;\;\;\;\;\;\;\;\;\;\;\;\;\;\;\;\;\;\;\;\;\;\;\;\;\;\;\;\;\;\;\;\;\;\;\;\;\;\times \text{Pr}(\mathrm{present}|\mathrm{sensed})+\text{Pr}(\bar{\mathrm{sensed}})\\ \;\;\;\;\;\;\;\;\;\;\;\;\;\;\;\;\;\;\;\;\;\;\;\;\;\;\;\;\;\;\;\;\;\;\;\;\;\;\;\;\;\;\;\;\;\;\;\;\;\;\;\times \text{min}({\mathrm{cost}}_{\mathrm{false positive}}\times \text{Pr}(\mathrm{absent}|(\bar{\mathrm{sensed}}),{\mathrm{cost}}_{\mathrm{false negative}}\\ \;\;\;\;\;\;\;\;\;\;\;\;\;\;\;\;\;\;\;\;\;\;\;\;\;\;\;\;\;\;\;\;\;\;\;\;\;\;\;\;\;\;\;\;\;\;\;\;\;\;\;\times \text{Pr}(\mathrm{present}|(\bar{\mathrm{sensed}})) \end{array}$$

EVOI thus corresponds with the difference between *E*(*cost_lower_*) and *E*(*cost_upper_*), where *lower* refers to the lower branch of the decision tree (*i.e.*, without sensor) and *upper* to the upper branch (*i.e.*, with sensor).

For mapping, however, we are concerned with a study area and aim to find the optimal additional sample locations as the new configuration that maximises global EVOI and thus minimises *E*(*cost_upper_*), *i.e.*, the accumulated expected misclassification costs. When mapping an area based on point observations, one considers observations not only to carry information on their locations but also about some neighbourhood. Points should thus not be considered in isolation because their joint distribution matters. The accumulated expected misclassification costs were computed by creating maps for each potential outcome $\left\{\mathrm{sensed},\bar{\mathrm{sensed}}\right\}$ at the set of new sensor locations using indicator kriging (see next section) and multiplying the expected costs for these situations with the joint probability of their occurrence.

Computation of this joint probability employed indicator kriging as well, within an iterative procedure. Writing *S*_1_ = *s*_1_ as shorthand for the outcome $\left\{\mathrm{sensed},\bar{\mathrm{sensed}}\right\}$ at a sensor location indexed by the number 1 and explicitly acknowledging the conditioning on previous data, the joint probability of outcomes at multiple locations was obtained using the probability chain rule (Equation (5)):

(5) $$\text{Pr}(\cap_{k=1}^{n}S_{k,\mathrm{now}}|\mathrm{previous data})=\prod_{i=1}^{n}\text{Pr}(S_{k,\mathrm{now}}|\cap_{l=1}^{n-1}S_{l,\mathrm{now}},\mathrm{previous data})$$

where $\cap_{k=1}^{n}S_{i,\mathrm{now}}$ denotes the joint outcome *S*_1_ = *s*_1_, ..., *S*_n_ = *s*_n_ and *n* is the number of sensor measurements. The iteration starts by computing Pr(*S*_1,*now*_|*previous data*), next Pr(*S*_1,*now*_, *S*_2,*now*_|*previous data*) = Pr(*S*_1,*now*_|*previous data*) × Pr(*S*_2,*now*_|*S*_1_, *previous data*), and so on. Of course, as soon as an intermediate conditional probability approaches 0, further computation of the probability of that joint outcome is useless, so it was stopped and calculation of the corresponding expected costs was skipped.

It can be easily seen that computational demands increase dramatically with the number of locations to be simultaneously optimised. For example, with two simultaneous observations, four expected cost maps and their probabilities need to be computed for each pair of measurement locations being evaluated while the solution space increases by a factor 0.5(*m*−1), with *m* being the number of potential sample locations (e.g., number of grid nodes). Nearly optimal solutions may be obtained within a fraction of the exhaustive computational cost using a search heuristic such as simulated annealing or a genetic algorithm.

### 2.2. Indicator Kriging

Indicator kriging is a pragmatic approach for mapping the probability that a random variable, say *Z* exceeds some defined threshold, which has its origin in the early 1980s [25,26]. The basic idea is that the original random variable is transformed into a set of variables which attain the values 0 or 1 depending on whether or not the corresponding cut-off value is exceeded. Next, conventional geostatistical methods [26–29] are used on the indicator variables, *i.e.*, semivariance models are determined for the indicator transformed data and the latter are linearly interpolated using some form of kriging. The semivariance model describes the spatial dependence structure of the indicator variable as a function of distance and direction (the latter if anisotropy is considered) while kriging is a linear spatial interpolator that explicitly accounts for spatial correlation among observations of the variable. We used ordinary kriging, which implies that the unknown spatial mean (*i.e.*, total area where the threshold is exceeded) was estimated from the data. After solving order-relation problems [26,27], *i.e.*, predicted indicators are outside the [0, 1] interval or indicators corresponding to successively higher thresholds do not increase monotonically, the distribution of the original variable is recovered. Criticism on indicator kriging includes the invalidity of using additive linear models on indicator transformed data [26]. Computational and methodological simplicity, however, contribute to its continuing popularity.

For our purpose, we defined a single threshold at the critical level of the pollutant. Therefore the order relation problem only concerned conformance to the [0, 1] interval. Resuming the notation used earlier and noting that we do not observe the phenomenon itself but rather measure its presence with a sensor that is prone to measurement error, the indicator transform was realized as expressed by Equation (6):

(6) $${\text{s}}_{\text{k}}=\{\begin{array}{l} 1,\text{if }z_{k}\geq \text{threshold},\text{ with probability }\mathrm{Pr}(\mathrm{sensed}|\mathrm{present});\text{else }0\\ 0,\text{if }z_{k}<\text{threshold},\text{ with probability }\mathrm{Pr}(\bar{\mathrm{sensed}}|\mathrm{absent});\text{else }1 \end{array}$$

where *z_k_* refers to the usually unobserved true state of the random variable *Z* at location *k*. So the data concern the probability of a positive sensor measurement while computation of (global) EVOI requires the probabilities of the true state of the phenomenon, Pr(*present*) and Pr(*absent*), see Equations (1)–(3). The nugget of the semivariogram—if modelled—represents both measurement error and short-range spatial variation, where sensor specifications may be used to identify both components. Model-based geostatistics allows distinguishing between measurements and the random variable of interest itself during the prediction stage, as discussed in [30] (p. 139).

For computation of global EVOI, the two possible states of *z_k_* at each sensor location were considered and these were combined with the previously measured data. So with *n* sensors there are 2*^n^* potential joint outcomes to be evaluated. Once the new sensor configuration had been selected by maximisation of global EVOI, new data were obtained by actually measuring the field representing reality through evaluation of Equation (6).

### 2.3. Regression Kriging with Indicator Data

Often, geostatistical interpolation is not only based on observations of the target variable; in case auxiliary data are available, hybrid interpolation techniques which combine different data sources can be used to improve prediction. If the auxiliary data exhaustively cover the study area, regression kriging [31,32] is a widely accepted option. In that case, first regression analysis is applied for fitting and predicting a general trend of the mean response of the target variable on the auxiliary data. In the second step, local deviations from the trend are interpolated by simple kriging, with zero mean and using a semivariogram of the residuals between the regression response and the data. The final prediction is the sum of the regression response and the simple kriging results. Details can be found in [31]. Regression kriging readily accommodates generalised linear models, as is needed when dealing with indicator data which are bounded to the [0, 1] interval. For these data logistic regression [33] is the method of choice.

### 2.4. Spatio-Temporal Kriging

Space-time geostatistics enable data analyses and prediction by taking into account the joint spatial and temporal dependence between observations [34–36]. From a practical point of view, which is also supported by theory [34], the temporal dimension can be interpreted simply as an extra dimension to be included in the distance metric which is used in the semivariance structure. If the spatial, temporal and combined spatio-temporal components of the data generating process are assumed stationary and mutually independent, the sum-metric semivariance structure is obtained [34,36]:

(7) $$\gamma (h_{s},h_{t})=\gamma_{s}(h_{s})+\gamma_{t}(h_{t})+\gamma_{st}(\sqrt{{h_{s}}^{2}+\alpha {h_{t}}^{2})}$$

where *γ* denotes the semivariance, *h* is a lag distance, the subscripts *s* and *t* refer to space and time respectively and *α* is a geometric anisotropy ratio a that is needed because of differences between the units of distance in space and time. We used this approach in the dynamic case study, which is detailed in Section 3.2.

## 3. Case Studies

### 3.1. Static Field

Pollution of the environment by, for example, deposited radionuclides or polyaromatic hydrocarbons after some calamity can be represented by a static field if autonomous changes to the system are slow in comparison to the length of the measurement campaign and subsequent management of the problem. To illustrate the EVOI approach on a static field, a synthetic data set was constructed by applying a threshold at 20, say ppm, to a stationary Gaussian random field of 100 × 100 grid cells of unit size with mean 20 (ppm) nugget 1 (ppm^2^) representing short range variability and an isotropic spherical structural spatial correlation component with range 40 spatial units and a partial sill (semivariance) 16 (ppm^2^). Sensor data were simulated by sampling the synthetic data (Equation (6)), with Pr(*sensed* | *present*) = 0.98. The initial sample consisted of 16 points on a regular grid. Sensor data were interpolated by ordinary indicator kriging assuming a spherical indicator semivariogram without nugget, a range of 20 spatial units and a sill of 0.25 (no unit). Notice that (1) the semivariogram differs from the one that generated the data and (2) measurement errors were considered negligible at the prediction stage. All computations were performed in R [37,38] using the geostatistical package *gstat*[29].

Three scenarios were considered for adding new measurement locations to the original sample:

1. add a single measurement at a time at the location leading to the highest global EVOI, moving the sensor with the lowest cost (in this case Euclidean distance);
2. select two sensors and add measurements from two locations simultaneously by scanning the area that can be reached by each sensor within a single time step. Again the solution leading to the highest global EVOI is chosen at each time step;
3. add two sample locations simultaneously by scanning the complete area for the highest global EVOI and move the sensors with lowest cost distance. To speed up computations, a genetic algorithm, *i.e.*, the package *genalg* for R [37]) was used.

The costs of misclassification were arbitrarily set at 2 and 3 cost units for false positives and false negative, respectively. As indicated above and similar to other work [11,12,15,16,18], the required semivariogram models were assumed to be given at the start of the adaptive part of the survey. Under practical circumstances such a situation may arise when the semivariogram is estimated from the initial sample or using data from an earlier comparable survey.

Maps generated by EVOI optimisation (scenario 1) were compared with maps interpolated using measurements obtained by: (1) random sampling and (2) sample locations determined by minimisation of the kriging variance [12]. To that end, 100 binary fields were simulated using the procedure described above but—to avoid confounding effects—assuming perfect sensors, *i.e.*, $\text{Pr}(\mathrm{sensed}|\mathrm{present})=\text{Pr}(\bar{\mathrm{sensed}}|\mathrm{absent})=1$. The true accumulated misclassification costs after adding 16 additional measurements were computed by comparing the final predicted maps with the corresponding true binary field on a pixel-by-pixel basis and multiplying any error with the respective costs of misclassification. Differences between the mean misclassification costs were tested by *t*-tests of paired differences. Furthermore, measurement scenarios (2) and (3) were compared on the basis of expected and true accumulated misclassification costs.

### 3.2. Dynamic Plume

A dynamic plume of some pollutant which affected a 400 × 400 m area of Wageningen University campus was simulated. The plume was composed of a deterministic part, *i.e.*, a point source Gaussian plume which was rotated in a sinusoidal fashion with a peak amplitude of 0.1π radians and a period of 4 h. The Gaussian plume was simulated assuming a pollutant release of 30 kg/s at an effective height of 350 m above the ground and a wind speed of 5 m/s. A stochastic component representing both background levels and random deviations from the deterministic model was added to the waving plume. This component consisted of a spatio-temporal Gaussian random field with a mean of 50 ppm and a semivariance structure as detailed in Table 1. The critical level was set at 65 ppm at any moment in time (*i.e.*, no dose but an instantaneous level). This implies that the stochastic component contributed substantially to the total contaminant levels in the study area.

The deterministic part of the plume (thus excluding the stochastic deviations) was assumed to be given at the prediction stage. Accordingly, the deterministic plume was available as an auxiliary data source to support mapping presence/absence of the dynamic plume. To this end regression kriging with logistic regression on the deterministic plume was employed. Though methodologically feasible, no other explanatory variables were used in the regression model. For practical reasons, the regression coefficients were assumed to be static; they were determined just once based on the initial state of the true plume using a sample of 441 observations on a regular grid. The regression coefficients were determined by maximum likelihood. Residual variation was modelled by a spatio-temporal Gaussian random field.

For predicting the spatio-temporal residuals, the response of the logistic regression model of the corresponding moment in time was subtracted from realised previous measurements and potential current measurement outcomes. The interpolated residuals were subsequently added to the logistic response and truncated to the interval [0, 1] to compute the probabilities of presence and absence. Table 2 lists the parameters of the semivariance structure assumed at the prediction stage.

Previous observations of spatio-temporal residuals contain information on the next state since by construction the residual field is spatially and temporally correlated. However, unlike the static case, repeated measurement at the same location adds information to the system, since the temporal correlation is less than 1. With *n* sensors in the area and *m* potential locations to be visited there are $\left(\begin{array}{c} m\\ n \end{array}\right)$ unique sensor configurations. For example, with 16 sensors and a potential sample location every on a grid with a node spacing of 18 meters over the study area, there are $\left(\begin{array}{c} 441\\ 16 \end{array}\right)=7.426995\text{e}+28$ possible combinations of sensor locations. Each of these configurations has 2^16^ = 65536 potential outcomes of the sensor measurements, which renders exhaustive search over all possibilities prohibitively expensive. In this example, search space was substantially reduced by allowing only a single measurement location per time step to be changed; the other 15 sensors remained stationary. For each of the 16 sensor locations of the previous time step we tested alternative locations and the configuration having the lowest accumulated expected misclassification costs was selected. Initial sensor locations were again chosen on a regular grid.

The true misclassification costs accumulated over time and space achieved by EVOI sampling were compared with those obtained by:

1. each time step randomly selecting one sensor and measuring at a single randomly selected vacant location. The other 15 sensors stay and measure at their previous location (Random1);
2. random relocation of all sensors at each time step (Random16);
3. repeated measurement at the initial regularly spaced sample locations (Fixed).

The random sampling methods (1 and 2) were repeated 1,000 times.

## 4. Results and Discussion

### 4.1. Static Field

**Figure 2.**
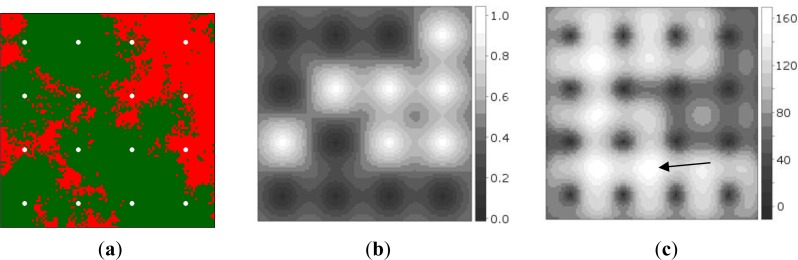
Static field. (**a**) Presence (red), absence (green) and initial sample (white dots). (**b**) Probability of presence; the point at the third row from above and third column from the left was incorrectly measured (random measurement error). (**c**) Global EVOI; the arrow points to the location having highest global EVOI. Probability and EVOI were computed from the initial sample of 16 regularly spaced points (a).

Figure 2(a) shows the synthetic data, while Figure 2(b) shows the probabilities of presence interpolated from the initial sample of 16 sites. It can be observed that measurement of the point at the second row from below and third column from the left produced a random measurement error value when Equation (6) was evaluated. Figure 2(c) shows the map of global EVOI, *i.e.*, EVOI computed after aggregating expected misclassification costs for observations made at each grid location, separately. The best location thus corresponds to the highest global EVOI. Not surprisingly, this occurs between observations differing in value (indicated by the arrow). If minimisation of kriging variance would have been used as the optimisation criterion, site selection would have been independent of the measured values.

**Figure 3.**
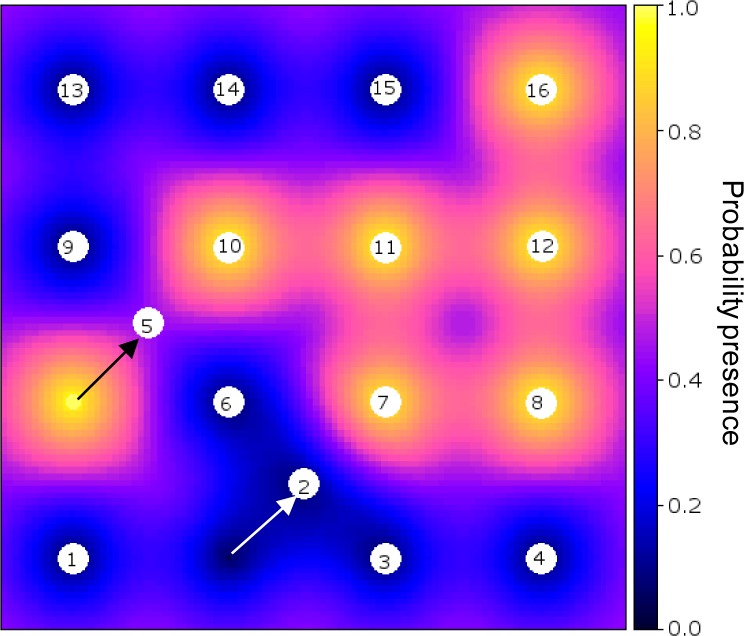
Configuration of initially regularly spaced sensors after two iterations with a single observation per step (scenario 1). First sensor 2 moved (white arrow) and a measurement was made, next sensor 5 moved (black arrow) but the measurement has not yet been made.

Figure 3 shows an example of an optimised sensor configuration after the 17th observation was made (16 initial and 1 infill measurements) on a backdrop of the probability of presence of the phenomenon (*cf*. Figure 2(b)).

Euclidean distance was used for deciding which sensor to move to the next location, but another cost criterion could have been used with only minor modification of the algorithm, as was demonstrated in [17].

**Figure 4.**
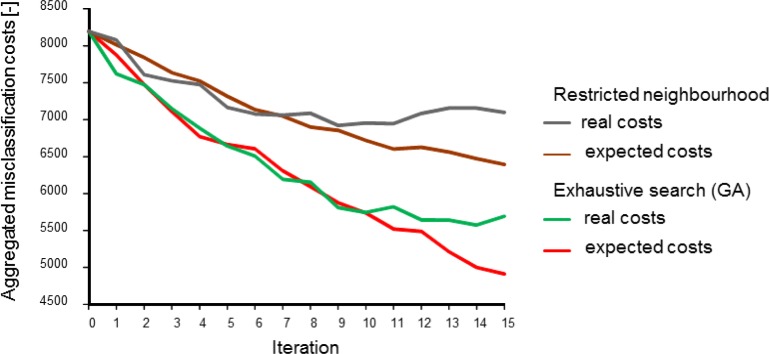
Effect of the way sensor constraints are taken into account on aggregated misclassification costs with two simultaneously moving sensors (scenarios 2 and 3).

**Figure 5.**
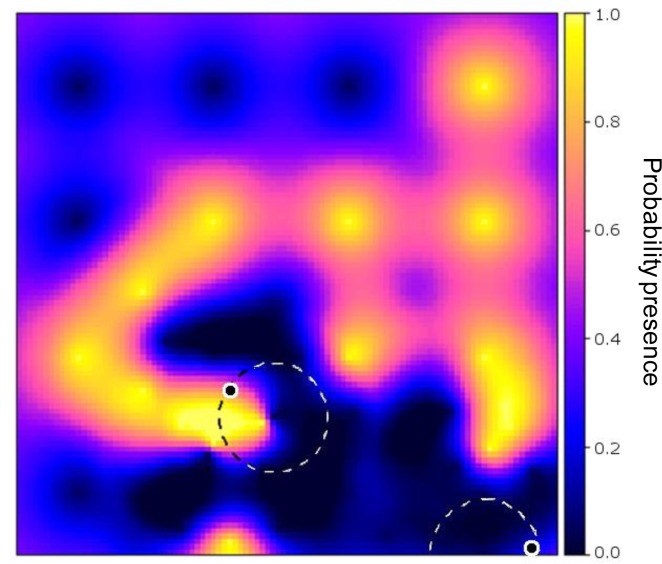
Probability of presence after 15 time steps in which two simultaneous sensor measurements were added (scenario 2). Each sensor only scanned a limited neighbourhood around their current position (indicated by the dashed circle) for the optimal solution, which caused them to get trapped. The concentric black-white dots (eyes) indicate the newly selected locations.

Figure 4 shows the effect of the two methods to account for sensor constraints as described in Section 3.1 (scenarios 2 and 3), with two simultaneously moving sensors. Not surprisingly, both the expected and the real misclassification costs were lower when the full study area was scanned in search of the best sample locations. In the alternative scenario, the sensors got trapped in an initially identified local optimum, as shown in Figure 5, where the area in the upper left will not be found by the sensors.

While the results obviously depend on the choice of the start locations, the figure exemplifies that one has to be careful in focusing on sensor constraints for planning adaptive sampling. In doing so, highly informative sites may never be visited because they are hidden behind data from earlier measurements. In contrast, a global search will identify those relevant sites and, next, sensor constraints may be used to find a strategy for reaching their locations.

Differences between real costs (usually not known) and expected costs (see the right-hand side of Figure 4) are indicative of misspecification of the geostatistical model used for computing the probabilities used in Equations (1) and (5). In the global search scenario, the true misclassification costs shown in Figure 4 suggest that there was no point in continuing the survey after ten observations had been added to the initial sample while the expected misclassification costs still decreased. The opposite can happen as well; between eleven and twelve measurements the expected costs stabilised while the true costs dropped. Obviously, when mapping a phenomenon in the real world, the true misclassification costs are unknown and thus there are no means for comparing real and expected misclassification costs. However, newly acquired data can be used to revise the geostatistical model and its parameters and hence improve predictions made by the model. The above demonstrates the importance of the choice and the parameter settings of the geostatistical model and it also advocates cautious interpretation of EVOI as a stopping criterion of a field survey.

Table 3 lists the reduction of true misclassification costs of the EVOI approach in comparison to random sampling and minimisation of the kriging variance after adding 16 measurements to first phase samples. The results were obtained based on 100 realisations of a Gaussian random field. Even if the mean improvements were small, they were all significantly better than 0 at the α = 0.05 level. During the experiment we observed that improvements increased with the number of measurements made [17]. This is likely to be caused by the relatively small sample (size = 16) of the first phase; during the second phase, initially any location is likely to add information to the system. Later, when the sample obtained thus far already holds substantial information, it really matters where to locate a new measurement because measurements at suitable locations will provide new information whereas poorly chosen sites will hardly or not at all. The relatively small improvement with respect to the kriging variance criterion is no surprise since the latter tends to fill data holes while random sampling may allocate measurements anywhere on the map.

### 4.2. Dynamic Plume

**Figure 6.**
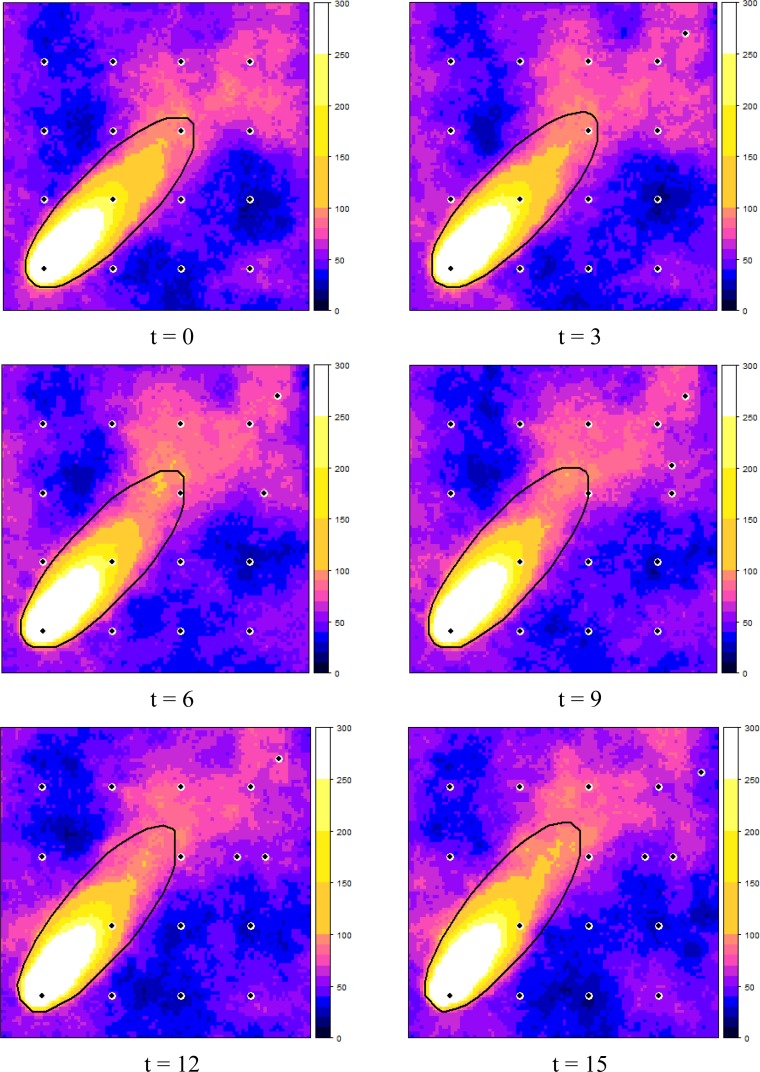

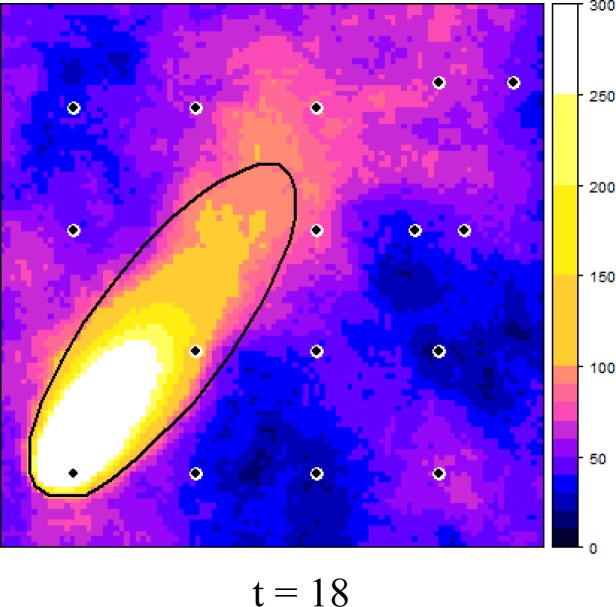
EVOI sampling of a dynamic plume (ppm), with single sensor relocation per time step. The black line delineates the critical level for the deterministic plume; black/white dots are the sensor locations.

Figure 6 shows how the dynamic plume and the selected measurement locations evolved over time, starting at t = 0, in six time steps of three minutes each. It can be observed that the EVOI approach moved the sensors downwind of the predicted plume. Intuitively, this makes sense and it is caused by the combined effect of (1) the relatively large contribution of the stochastic deviations from the deterministic plume and (2) the ratio of 1.5 between the costs of false negatives (costs = 3) and false positives (costs = 2). With less uncertainty, selected locations would tend to cluster near the boundary of the predicted plume (the green dots in Figure 6); with a cost ratio < 1 new observations would be located more towards the inside of the deterministic plume. Similar effects were also observed in [16].

No attempts were made to optimise the computer code, which resulted in a run time of several days to calculate relocation of a single sensor over the six time steps. We are well aware that this is far from realistic and therefore suggest the following improvements:

1. re-utilisation of the kriging weights during the analysis of the possible measurement outcomes of a sensor configuration. This is feasible because kriging weights are independent of the measured values, they only depend on the spatio-temporal configuration of data points;
2. using a search heuristic rather than exhaustive search, see also Section 3.1;
3. using dedicated compiled software rather than a script running in R.

Particularly suggestion (2) requires further research which should include finding appropriate parameter settings for the optimiser. A suitable search algorithm should also allow for concurrent site selection for multiple sensors. As explained in Section 3.2, this greatly affects the complexity of the problem.

**Figure 7.**
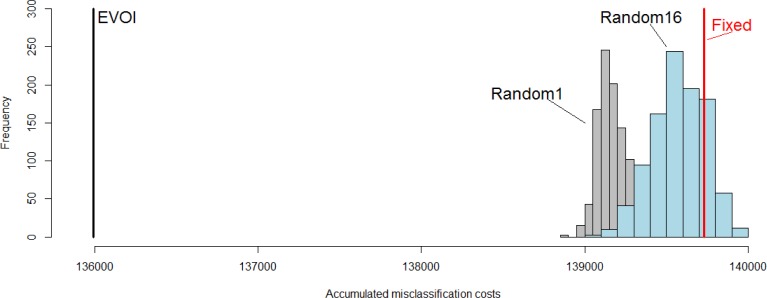
Comparison of the accumulated real misclassification costs of four sampling approaches. Distributions for Random1 and Random16 were obtained from 1,000 realisations.

EVOI sampling led to lower accumulated real misclassification costs (1.360e+5) than any of the alternative considered, as can be observed in Figure 7. Neither of the two random sampling approaches produced even a single realization having lower misclassification costs accumulated over space and time than EVOI sampling. Figure 7 also shows that the sensor configuration fixed at the initial locations was less accurate (misclassification costs = 1.397e+5) than the averages of the two random methods (1.392e+5 and 1.396e+5 for Random1 and Random16, respectively). Exploring locations beyond the initial configuration reduced the misclassification costs, but completely random sampling (Random16) was on average inferior to having only a single sensor exploring vacant sites each time tep (Random1). Due to computational complexity this study is inconclusive as to how many, where and when sensors should have remained at fixed locations according to the EVOI criterion for the case studied. We explained before that this complex problem calls for a suitably parameterized heuristic optimizer, which is left for future research. Once this is solved, future research could also explore alternative and typically more computationally intensive geostatistical models (see e.g., [30]).

## 5. Conclusions

The expected value of information (EVOI) approach allocates new observations at locations that intuitively make sense. Moreover, comparison with random sampling and sampling aiming for minimum kriging variance showed that the expected misclassification costs were significantly reduced with EVOI-sampling. The method accounts for data values and specified misclassification costs. The latter can be dissimilar for different kinds of errors (*i.e.*, false positives and false negatives). This way, site selection is directly affected by information about the spatio-temporal field as it becomes available as well as by the decision problem at hand and.

Constraining potential sample locations to the space that can be travelled by a small set of mobile sensors is a flawed strategy since the sensors may get trapped in some area and may thus fail to visit highly informative spots that are screened by previous observations. A better approach would be to first perform a global search for the highest EVOI and next use sensor constraints for deciding which sensors to move to the selected measurement sites.

With the help of indicator kriging, computation of EVOI for a given set of sample locations is computationally inexpensive and methodologically simple. Finding the optimal sensor locations, however, remains a very demanding task. This particularly holds when selecting multiple locations simultaneously such as in case of monitoring a dynamic spatial field. Meta-heuristic optimisers including genetic algorithms and simulated annealing may be useful for these situations, but this requires research beyond the scope of the current paper.

In this work, parameter uncertainty and uncertainty about the geostatistical model were not taken into account. However, divergence between the true and the expected misclassification costs after adding several measurements indicates that the approach is sensitive to model misspecifications. This may be consequential, for example if EVOI is used for deciding whether or not to stop a survey. Model parameterisation and dealing with uncertainty in the geostatistical model are therefore other aspects requiring further research.

## Figures and Tables

**Table 1.** Semivariance structure of the Gaussian random field added to the deterministic plume.

**Component**	**Shape^a^**	**Sill (ppm^2^)**	**Range**	**α (m/min)**
γ*_s_*	Sph	200	320 m	-
γ*_t_*	Sph	50	35 min	-
γ*_st_*	Sph	50	150 m	7.14

a “Sph” denotes a spherical shape.

**Table 2.** Semivariance structure used for predicting residuals for the dynamic plume.

**Component**	**Shape^a^**	**Sill (-)**	**Range^b^**	**α (m/min)**
γ*_s_*	Exp	0.085	33 m	-
γ*_t_*	Exp	0.025	12 min	-
γ*_st_*	Exp	0.025	50 m	7.14

a “Exp” denotes an exponential shape;

b Range parameter: practical range is approx. 3 × the listed value.

**Table 3.** EVOI improvements with respect to random sampling and minimisation of kriging variance.

**Sampling Approach**	**Mean Improvement (%)**	**Standard Deviation (%)**	***Pr*(*obs.*> 0 |*true*= 0)**
Random	7.1	12.0	2.4e−08
Min. kriging var.	3.0	9.9	1.6e−03
